# *Parvimonas micra*, *Peptostreptococcus stomatis, Fusobacterium nucleatum* and *Akkermansia muciniphila* as a four-bacteria biomarker panel of colorectal cancer

**DOI:** 10.1038/s41598-021-82465-0

**Published:** 2021-02-03

**Authors:** Muhammad Afiq Osman, Hui-min Neoh, Nurul-Syakima Ab Mutalib, Siok-Fong Chin, Luqman Mazlan, Raja Affendi Raja Ali, Andee Dzulkarnaen Zakaria, Chai Soon Ngiu, Mia Yang Ang, Rahman Jamal

**Affiliations:** 1 UKM Medical Molecular Biology Institute (UMBI), UKM Medical Centre, Universiti Kebangsaan Malaysia, Jalan Yaa’cob Latiff, Bandar Tun Razak, Cheras, 56000 Kuala Lumpur, Malaysia; 2 Department of Surgery, Faculty of Medicine, Universiti Kebangsaan Malaysia, Kuala Lumpur, Malaysia; 3 Department of Medicine, Faculty of Medicine, Universiti Kebangsaan Malaysia, Kuala Lumpur, Malaysia; 4 Department of Surgery, School of Medical Sciences, Universiti Sains Malaysia, Kubang Kerian, Kelantan Malaysia

**Keywords:** Cancer, Microbiology, Molecular biology

## Abstract

Dysbiosis of the gut microbiome has been associated with the pathogenesis of colorectal cancer (CRC). We profiled the microbiome of gut mucosal tissues from 18 CRC patients and 18 non-CRC controls of the UKM Medical Centre (UKMMC), Kuala Lumpur, Malaysia. The results were then validated using a species-specific quantitative PCR in 40 CRC and 20 non-CRC tissues samples from the UMBI-UKMMC Biobank. *Parvimonas micra*, *Fusobacterium nucleatum*, *Peptostreptococcus stomatis* and *Akkermansia muciniphila* were found to be over-represented in our CRC patients compared to non-CRC controls. These four bacteria markers distinguished CRC from controls (AUROC = 0.925) in our validation cohort. We identified bacteria species significantly associated (cut-off value of > 5 fold abundance) with various CRC demographics such as ethnicity, gender and CRC staging; however, due to small sample size of the discovery cohort, these results could not be further verified in our validation cohort. In summary, *Parvimonas micra*, *Fusobacterium nucleatum*, *Peptostreptococcus stomatis* and *Akkermansia muciniphila* were enriched in our local CRC patients. Nevertheless, the roles of these bacteria in CRC initiation and progression remains to be investigated.

## Introduction

Colorectal cancer (CRC) is the third leading cause of cancer-related deaths^1,2^. Emerging evidence indicates that dysbiosis of the gut microbiome is associated with the pathogenesis of CRC. Several studies have suggested the involvement of bacteria genera such as *Fusobacterium, Bacteroides, Parvimonas, Peptostreptococcus* and *Streptococcus* in colorectal carcinogenesis^3^. Interestingly, there also appears to be a geographical link in terms of the dominant species of the gut microbiome in CRC patients. While *Fusobacterium nucleatum* features as the common pathogen reported in many CRC studies around the world^4–7^, species such as *Peptostreptococcus assaccharolytica* (Canada and USA), *Granulicatella sp.* (Guangzhou, Hongkong and China) and *Collinsella sp.* (Netherlands) appear to be region-specific^7–9^. In Malaysia, a country situated in Southeast Asia, CRC is currently the second most common cancer in both men and women^10^. Malaysia is a developing nation with 3 major ethnic groups namely, Malay, Chinese and Indian. Chinese Malaysians has the highest incidence of CRC, nevertheless, the cause of it is still unknown^10,11^. Even though gut microbiome profiling studies have been performed in many countries, data from the South-East Asian region are still lacking. In this study’s discovery phase, we performed 16S rRNA gene sequencing to profile the mucosa-associated gut microbiome of Malaysian CRC patients. We then validated five microbial candidates found to be over-represented in CRC patients in the discovery cohort, using a qPCR assay. We also evaluated the sensitivity and specificity of the proposed CRC-associated gut microbiome panel to determine microbial signatures that are potentially specific for Malaysian CRC patients.

## Results

### Sample collection and subject demographics

A total of 36 subjects (newly diagnosed CRC patients, n = 18; healthy controls, n = 18) were enrolled for the discovery phase of the study. Demographic distribution between the subjects for both CRC and control groups were similar in terms of age, sex and ethnicity. For CRC patients, majority of the tumours were located at the left-sided colon (83.3%) and of Dukes’ B stage (61.1%). Non-CRC subjects were observed to be patients who attended the clinic for exploratory colonoscopy screening due to symptoms such as abdominal pain, altered bowel habit or family history of CRC. The demographic characteristics of the discovery cohort is shown in Table 1.

**Table 1:** Demographics of discovery phase study subjects.

Demographic	Cancer (n = 18)		Control (n = 18)	*p*-value
**Age (mean ± SD)**		64.88 ± 2.34	54.44 ± 2.91	
< 60 years (n, %)		7 (38.8)	9 (50.0)	0.738
> 60 years (n, %)		11 (61.2)	9 (50.0)	
**Gender (n, %)**				
Male		12 (66.7)	11 (61.1)	0.999
Female		6 (33.3)	7 (39.9)	
**Race (n, %)**				
Malay		9 (50.0)	11 (61.1)	0.784
Chinese		8 (44.4)	6 (33.3)	
Indian		1 (5.6)	1 (5.6)	
**Pathological staging (n, %)**				
Dukes’ B		11 (61.1)	N.A	
Dukes’ C		5 (27.8)	N.A	
Dukes’ D		2 (11.1)	N.A	
**Tissue location (n, %)**				
Left-sided		14 (77.8)	18 (100.0%)	
Right-sided		4 (22.2)	0 (0.0%)	

For the validation cohort, 40 CRC tissue samples and 20 control samples (18 from discovery cohort, two from UMBI-UKMMC Biobank) were included into the study. Tissues for all CRC stages were available for this part of the study, and the majority were of the Dukes’ C stage (57.5%). Similar to the discovery phase, most tumours were located at the left-sided colon (90.0%). The demographic characteristics of the validation cohort is shown in Table 2.

**Table 2:** Demographics of validation phase study subjects.

Demographic	Cancer (n = 40)	Control (n = 20)	*p*-value
**Age (mean ± SD)**	67.90 ± 1.53	55.50 ± 2.872	
< 65 years (n, %)	13 (32.5)	9 (45.0)	0.105
> 65 years (n, %)	27 (67.5)	11 (55.0)	
**Gender (n, %)**			
Male	22 (55.0)	13 (65.0)	0.584
Female	18 (45.0)	7 (35.0)	
**Race (n, %)**			
Malay	18 (45.0)	11 (55.0)	0.237
Chinese	22 (55.0)	8 (40.0)	
Indian	–	1 (5.0)	
**Pathological staging (n, %)**			
Dukes’ A	2 (5.0)	N.A	
Dukes’ B	12 (30.0)	N.A	
Dukes’ C	23 (57.5)	N.A	
Dukes’ D	3 (7.5)	N.A	
**Tissue location (n, %)**			
Left-sided	36 (90.0)	20 (100.0%)	
Right-sided	4 (10.0)	0 (0.0%)	

### Altered mucosal microbiome landscape in CRC

**Figure 1:**
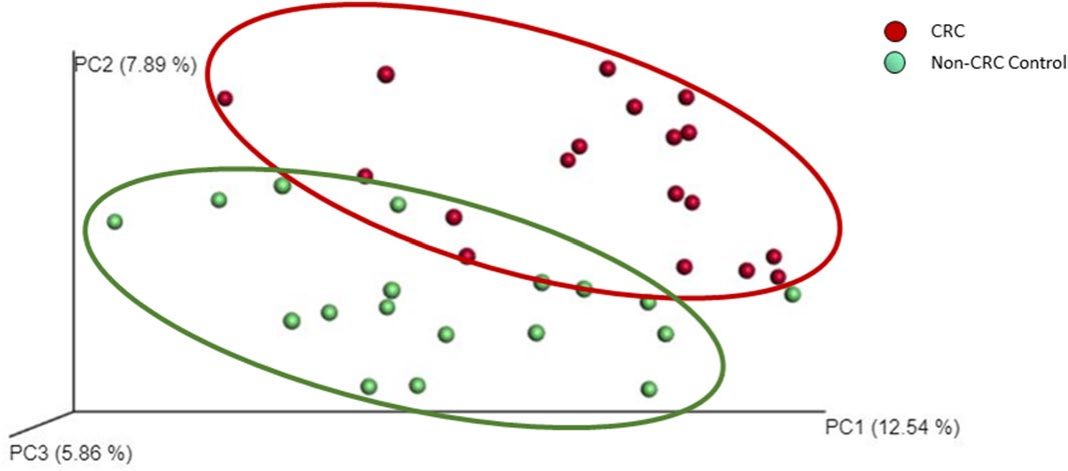
β-diversity of gut microbiome in CRC patients compared to non-CRC controls. Principal coordinates analysis (PCoA) plot of gut microbiome β-diversity in the subjects of this study based on unweighted UniFrac analysis.

16S rRNA gene sequencing generated a total of 7,940,453 high quality reads (mean ± SD ~ 220,568 ± 144,691) for all samples of the discovery cohort. Paired-end reads were clustered into Operational Taxonomic Units (OTU) at 97% similarity and taxonomic classifications were assigned to bacterial genera. From our core diversity analysis, we identified a total of 21 bacterial phyla and 358 genera for all included samples. Comparison of alpha diversity abundance revealed significant difference between the mucosal microbiome architecture of CRC patients compared to controls. Principal coordinate analysis (PCoA) plot of unweighted UniFrac analysis highlighted distinct β-diversity microbiome clusters (Fig. 1). Overall, mucosal microbiome composition differed significantly between CRC and non-CRC control subjects according to unweighted UniFrac distance.

**Figure 2:**
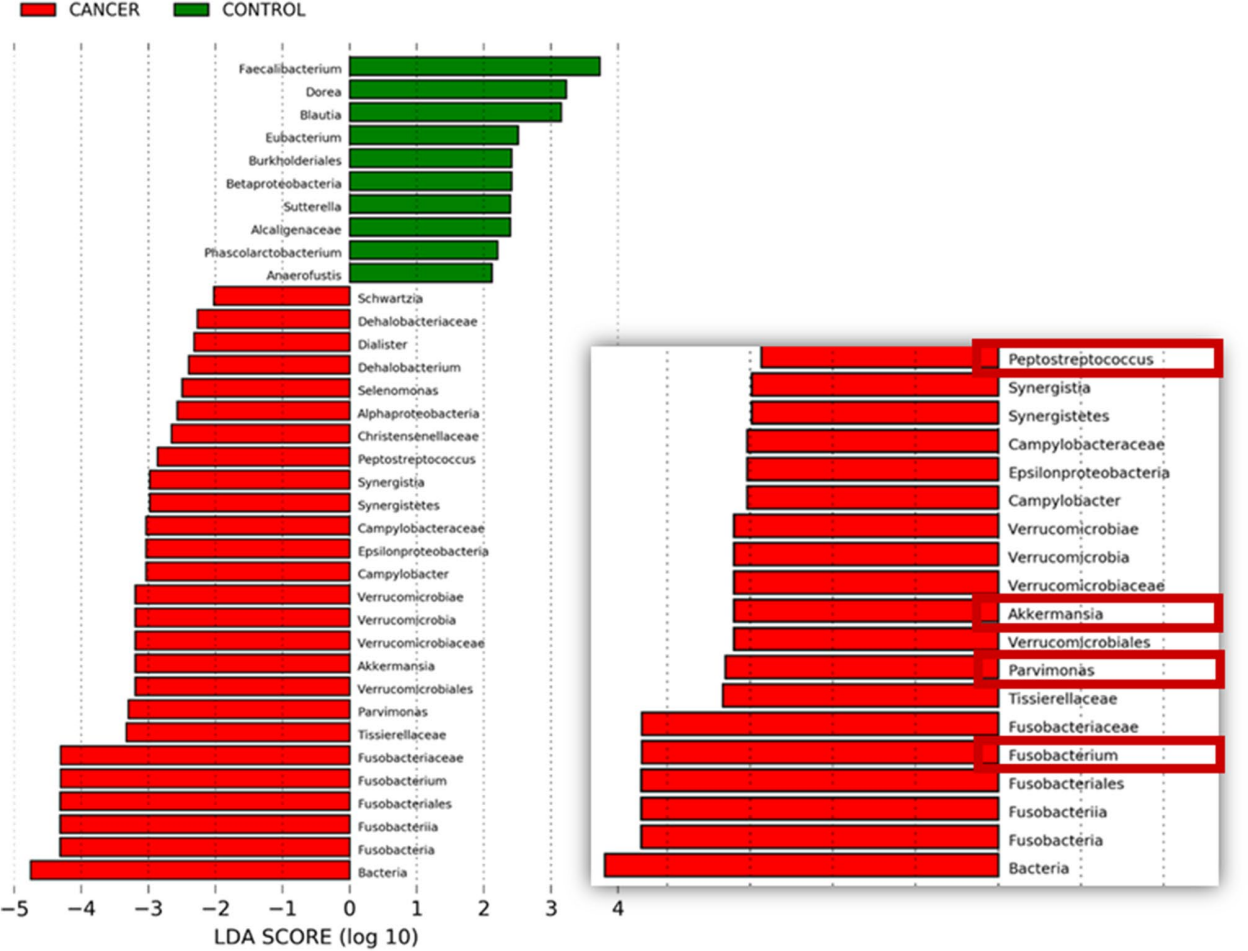
LEfSe analysis of bacterial taxa in CRC patients compared to non-CRC controls. LEfSe applies a Kruskal–Wallis rank-sum test, Wilcoxon rank-sum test, and linear discriminant analysis to determine the biological relevance of significantly-enriched taxa and ranks them by effect size. LDA score shows the magnitude of the effect size.

At the phylum level, patients with CRC were found to harbour increased abundance of *Fusobacteria, Verrucomicrobia* and *Synergitetes*; while nine genera found to be significantly enriched in CRC were *Filifactor, Prevotella, Peptostreptococcus, Akkermansia, Parvimonas, Lachnobacterium, Bulleidia, Dialister* and *Fusobacterium*. Interestingly, *Faecalibacterium, Dorea, Sutterella, Propionibacterium, Neisseria* and *Anaerofuctis* were significantly depleted in CRC patients (*p* < 0.05). Over-representation of *Fusobacterium nucleatum, Intestinimonas butyriciproducens, Peptostreptococcus stomatis, Eubacterium coprostanoligenes, Ruminococcus bromii, Bacteroides fragilis, Akkermansia muciniphila*, *Ruminococcus callidus, Parvimonas micra,* and *Gemella morbillorum* was found in more than 66% of CRC patients. In contrast, *Haemophilus parainfluenzae, Atopobium parvulum* and *Clostridium oroticum* were significantly depleted in CRC patients. On the other hand, we also found several novel CRC-related bacteria species such as *Intestinimonas butyriproducens, Eubacterium coprostanoligenes* and *Ruminococcus bromii* which have yet to be associated with CRC. Table 3 shows top 12 bacterial species over-represented in CRC (> 1.5-fold, occurrence in > 66% CRC samples) patients compared to controls. Figure 2 shows LEfSe analysis of bacterial taxa in CRC and non-CRC control subjects. *Fusobacterium, Peptostreptococcus, Parvimonas* and *Akkermansia* were amongst bacteria genera enriched in CRC patients, indicating a state of dysbiosis of the mucosal microbiome architecture in CRC.

**Table 3:** Top 12 bacterial species over-represented in CRC compared to controls.

Taxa name	Fold change	*p-*value	Occurrence in CRC (%)	Occurrence in control (%)
*Gemella morbillorum*	8.567	0.002	66.7	5.5
*Peptostreptococcus stomatis*	4.429	< 0.001	83.3	33.3
*Akkermansia muciniphila*	3.308	0.001	72.2	33.3
*Fusobacterium nucleatum*	3.136	< 0.001	100.0	61.1
*Ruminococcus callidus*	3.087	0.007	72.2	22.2
*Parvimonas micra*	3.049	0.001	72.2	50.0
*Eubacterium coprostanoligenes*	3.047	0.002	77.8	27.7
*Solobacterium moorei*	2.814	0.035	66.7	27.7
*Christensenella timonensis*	2.716	0.021	66.7	33.3
*Intestinimonas butyriciproducens*	2.612	0.001	88.9	38.9
*Ruminococcus bromii*	2.081	0.026	77.8	38.9
*Bacteroides fragilis*	1.875	0.030	77.8	44.4

### CRC-associated bacterial species as potential biomarkers

**Figure 3:**
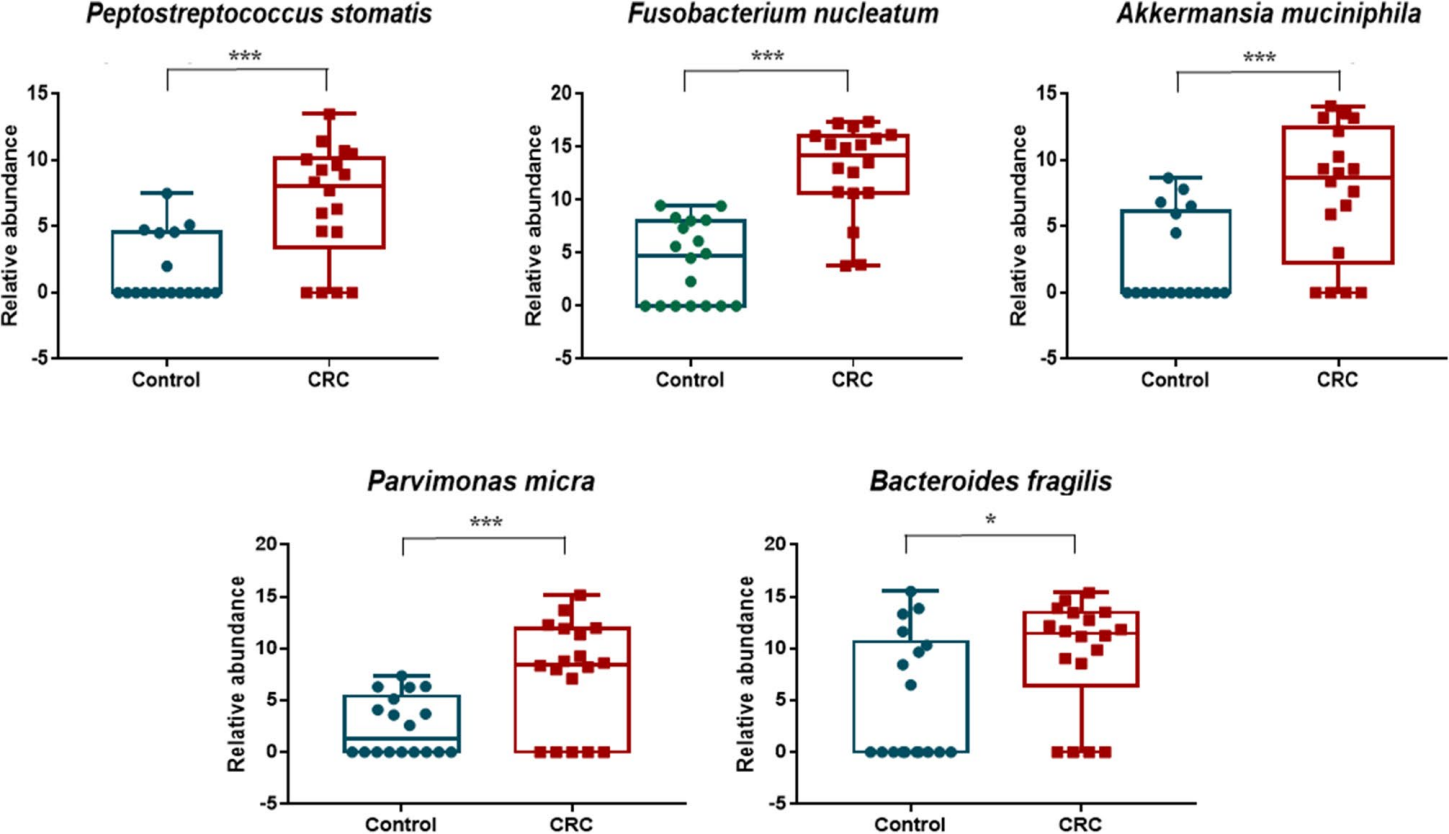
Gut bacteria identified to be over-represented in CRC patients compared to non-CRC controls. Boxplots show abundances of 5 bacterial species (*Fusobacterium nucleatum*, *Akkermansia muciniphila*, *Parvimonas micra*, *Peptostreptococcus stomatis* and *Bacteroides fragilis*) which were significantly over-abundant in CRC samples compared to non-CRC controls in the discovery cohort. Significance values: * < 0.05 and *** < 0.001.

From our list of 12 most over-represented bacterial species in CRC, whole genome sequences of *Gemella morbillorum, Ruminococcus callidus, Eubacterium coprostanoligenes, Solobacterium moorei* and *Intestinimonas butyriciproducens* are available; however, we encountered difficulty in primer design for these bacteria to validate their abundance via qPCR. This was mostly due to high similarity in regions suitable for primer design among different species of the same bacterial genus. Amplification of larger regions can differentiate these bacteria; however, qPCR products should be < 500 bp in size. In addition, at the time of this analysis, the whole genome sequences of *Christensenella timonensis* and *Ruminococcus bromii* are still not available. Therefore, validation of bacteria abundance was only carried out in five bacteria, namely, *Fusobacterium nucleatum* (Fn), *Akkermansia muciniphila* (Am), *Parvimonas micra (Pm), Peptostreptococcus stomatis* (Ps) and *Bacteroides fragilis* (Bf) (Fig. 3). Interestingly, bivariate correlation analysis demonstrated strong positive correlation (Spearman’s *r* = 0.85–0.918, *p* < 0.001) between 16S rRNA gene sequencing (discovery phase) and qPCR (validation phase) abundance for Fn, Am, Pm and Ps (Table 4). On the other hand, Bf qPCR abundance was found to be poorly correlated to 16S rRNA gene sequencing abundance.

**Table 4:** Correlation between bacterial candidate marker abundance detected from sequencing and qPCR.

Bacteria	Spearman’s rho (R)	*p*-value
*Fusobacterium nucleatum*	0.918	< 0.001
*Akkermansia muciniphila*	0.881	< 0.001
*Peptostreptococcus stomatis*	0.840	< 0.001
*Parvimonas micra*	0.815	< 0.001
*Bacteroides fragilis*	0.275	0.216

Samples with no reads in sequencing and Cq > 35 in qPCR were excluded from the correlation analysis.

We found all Fn, Am, Pm and Ps to show significant enriched abundance in the CRC patients of our validation cohort (*p* ≤ 0.001), where all patients had an abundance of more than 66% of these bacteria, while non-CRC controls had only less than 30%. Supplementary Dataset 1 shows qPCR data for both discovery (16S rRNA gene sequencing vs qPCR assay) and validation (validation cohort on qPCR assay) phase experiments.

**Figure 4:**
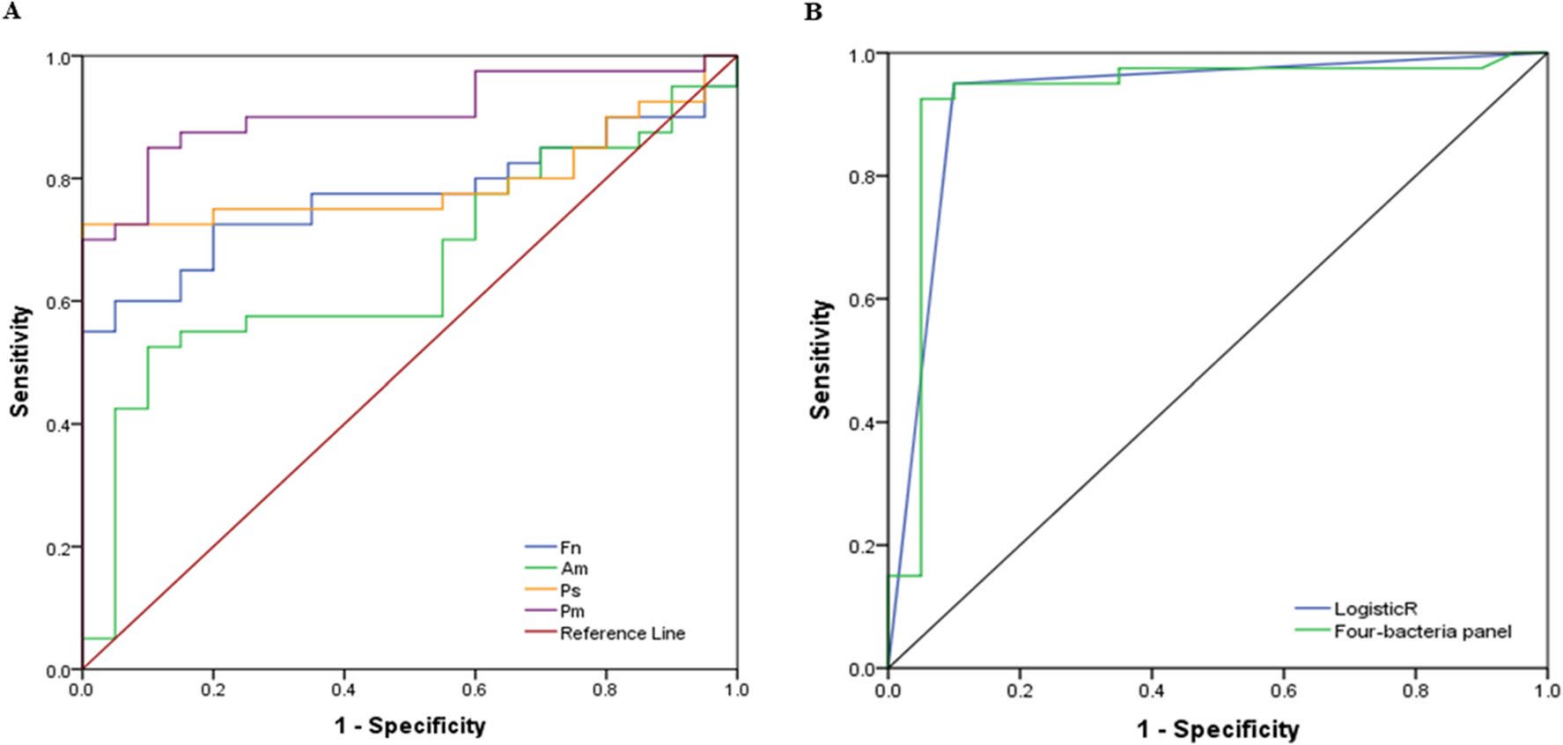
Diagnostics performance of the Pm-Ps-Fn-Am qPCR four-bacteria CRC biomarker panel. **(A)** ROC curves for Pm–Ps–Fn–Am in distinguishing CRC patients from non-CRC controls of the validation cohort. **(B)** ROC curves for the Pm–Ps–Fn–Am panel and probability plot values from the logistic regression (LogisticR) model.

Accordingly, the abundance of Bf was not found to be significantly different between CRC patients and controls (Table 5). Receiver Operating Curve (ROC) analysis (Fig. 3) showed that Pm had the best area under receiver operating curve (AUROC) of 0.908 (sensitivity, Sn = 85.0%, specificity, Sp = 90.0%; positive predictive value, PPV = 94.4%, negative predictive value, NPV = 75.0%) (Table 5). Combining these four bacteria as a CRC biomarker panel improved the indicative performance of these gut microbiota towards the occurrence of CRC, with an AUROC (ROC Test) of 0.927 (Sn = 95.0%, Sp = 90.0%, PPV = 95.0%, NPV = 90.0%) (Table 6). The predictive performance of this panel was further confirmed in LogisticR analysis (AUROC = 0.925) (Table 7 and Fig. 4).

**Table 5:** Enriched abundance of Pm, Ps, Fn and Am in CRC patients (validation cohort).

Bacteria	AUROC	*p*-value	95% confidence interval	
			Lower bound	Upper bound
*Parvimonas micra*	0.908	< 0.001	0.833	0.982
*Peptostreptococcus stomatis*	0.795	< 0.001	0.680	0.910
*Fusobacterium nucleatum*	0.771	0.001	0.654	0.888
*Akkermansia muciniphila*	0.657	0.048	0.517	0.798
*Bacteroides fragilis*	0.608	0.177	0.467	0.748

**Table 6:** Performance of Pm, Ps, Fn and Am as CRC biomarkers (validation cohort).

Variable	*Pm*	*Ps*	*Fn*	*Am*	Combination of *Pm, Ps, Fn* and *Am*
AUROC	0.908	0.795	0.771	0.657	0.927
Cut-off	0.0000716	0.0004165	0.0000834	0.0001880	0.0002450
Sensitivity	85.0%	72.5%	72.5%	55.0%	95.0%
Specificity	90.0%	100.0%	80.0%	80.0%	90.0%
PPV	94.4%	100.0%	87.9%	84.6%	95.0%
NPV	75.0%	64.5%	59.3%	47.1%	90.0%

*NPV* negative predictive value, *PPV* positive predictive value.

**Table 7:** Diagnostic performance of the Pm–Ps–Fn–Am four-bacteria CRC biomarker panel.

Statistical analysis	AUROC	P-value	95% confidence interval	
			Lower bound	Upper bound
ROC test	0.927	< 0.001	0.836	1.000
LogisticR	0.925	< 0.001	0.839	1.000

### PICRUSt analysis of CRC-associated gut microbiome

**Figure 5:**
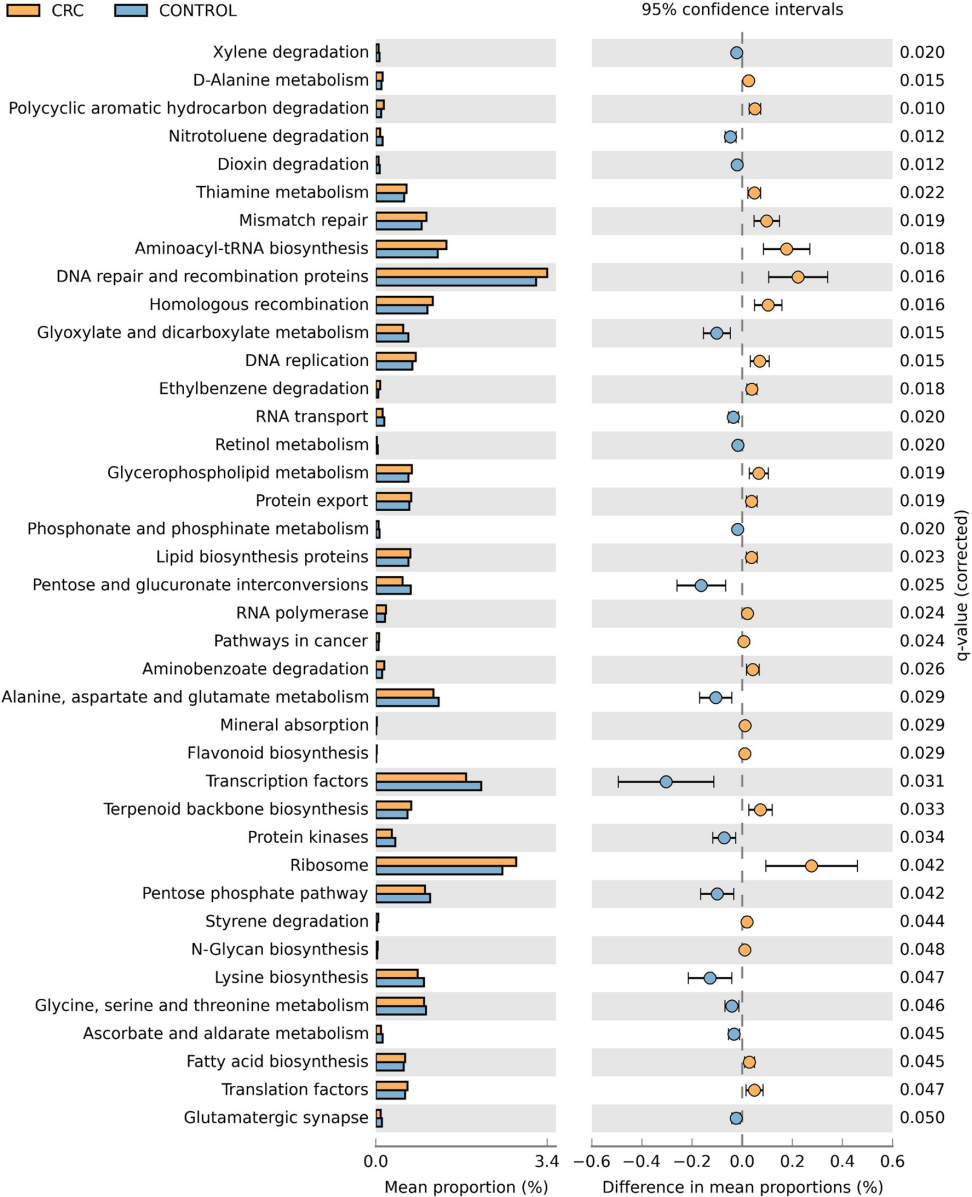
Predicted gut microbiome metabolic functions at KEGG pathway Level 3. PICRUSt and STAMP analyses revealed 39 significantly different metabolic functions based on gut microbiome abundance in CRC versus non-CRC controls.

Thirty-nine significant metagenome functions (KEGG Level 3) of both CRC and non-CRC control subjects’ gut microbiome were identified. Enriched metabolic functions of CRC gut microbiome were predicted to have roles in ribosome, DNA repair and recombination proteins, aminoacyl-tRNA biosynthesis and polycyclic aromatic hydrocarbon degradation. On the other hand, non-CRC gut microbiome metabolic functions involved pathways such as transcription factors, pentose and glucuronate interconversions, lysine biosynthesis and glyoxylate and dicarboxylate metabolism (Fig. 5).

### Taxonomic alterations of CRC-associated microbiome according to patient demographics

At a cut-off value of > 5 fold abundance*, Sporobacter termitidis, Ruthenibacterium lactatiformans* and *Akkermansia muciniphila* were found to be enriched in our Malay CRC patients, compared to *Harryflintia acetispora, Gemella morbillorum* and *Ruminococcus albus* in Chinese-Malaysian CRC patients. On the other hand, *Gemella morbillorum, Desulfovibrio desulfuricans* and *Eubacterium siraeumwere* were overrepresented in male CRC patients, compared to *Megasphaera elsdenii* in the females. *Eikenella corrodens* and *Eubacterium ventriosum* were found to be abundant in early-stage CRC patients, compared to *Prevotella intermedia, Harryflintia acetispora* and *Dialister pneumosintes* in advanced CRC patients. Over-abundant microbiome species according to various patient demographics is presented in Supplementary Dataset 2.

## Discussion

Gut microbiome dysbiosis is a hallmark of CRC. In our study, we profiled the gut microbiome of our study subjects, and observed respective clustering of CRC and non-CRC groups via unweighted UniFrac analysis. Interestingly, Pm, Ps, Fn and Am were further identified as the predominant bacteria associated with CRC locally. Bacteria found over-represented in our CRC patients were also reported in other geographical areas of the world^7,8,12^. Interestingly, our results are largely in concordance with a recently published report on a separate set of CRC patients recruited in another Malaysian hospital^13^, with the exception that Bf was not found to be consistently over-represented in our CRC patients. Bf*,* designated as the “driver” bacteria of CRC tumorigenesis via production of genotoxic molecules, are hypothesized to be one of the earliest colonizers on the colon mucosa of CRC patients^14^. These bacteria will be outcompeted by tumour-foraging opportunistic “passenger” bacteria such as Fn in subsequent stages of CRC. Incidentally, we had a lower number of early-stage CRC patients (Dukes’ A, n = 2, 3.4%, only in validation cohort) compared to that of the other Malaysian study (stage I, n = 5, 11.4%). Indeed, we did not manage to enrol any Dukes’ A patient in our discovery cohort; this might have also caused the poor correlation between sequencing (discovery) and qPCR (validation) results for Bf.

Our study identifies Fn as being commonly over-represented for all the demographic subtypes of the CRC patients in our study. The role of Fn in CRC tumorigenesis has been investigated and reported in many studies. The bacteria has been shown to induce tumorigenesis via Toll-like receptor 4 signalling to MYD88, causing initiation of inflammatory nuclear factor-kappa B (NF-kB) signalling pathway^4,15^. Fn has also been reported to adhere and invade into epithelial cells, stimulating the β-catenin pathway and causing activation of pro-inflammatory and oncogenic events^16^. On the other hand, the roles of Am, Pm and Ps in CRC tumorigenesis are still unclear. Intriguingly, even though Am has been reported to be reduced in patients with inflammatory bowel diseases^17^, it has been reported to cause tumorigenesis in mouse models ^18^. Pm and Ps are oral commensals which occasionally may turn into pathogens and have been reported to be associated with CRC. These two bacteria have been identified to be abundant in some CRC gut microbiome studies carried out in Canada, USA and China^7,8,19,20^.

In our study, Fn appears to be consistently over-represented in all CRC demographics, and high abundance (> 2 fold, data not shown) of Pm, Ps, Fn and Am (our CRC microbiome predictive panel) were observed across all cancer patients. Our findings suggest that over-abundance of these four bacteria could implicate colorectal carcinogenesis. Fn might have been integral in creating a pro-inflammatory environment and tumorigenesis of colon cells^4,15,16^. Meanwhile, Pm and Ps, which are oral microbiome and biofilm producers, secrete biofilm which protect cancer cells from the host’s immune system^21^. Am, where it has been recently reported to be positively correlated with the host’s immunotherapeutic response in mice, might be playing a role in reducing the pro-carcinogenesis effect of pathogenic bacteria such as Fn^22,23^. Nevertheless, the exact roles played by these four bacteria in CRC are still unknown, and remains to be investigated. Future experiments involving co-culture of these four bacteria with colon cells could be conducted to further investigate this. In a recent study, meta-analyses of the CRC microbiome were performed through comparisons across multiple datasets and populations, whereby *Fusobacterium, Porphyromonas, Parvimonas, Peptostreptococcus, Gemella, Prevotella*, and *Solobacterium* species were found to be enriched in CRC patients^24,25^. Another study which also performed multi-cohort analysis of CRC gut microbiome showed significant abundance of *B. fragilis*, *F. nucleatum*, *P. asaccharolytica*, *P. micra*, *P. intermedia*, *A. finegoldii*, and *T. acidaminovorans* in CRC subjects^26^. Interestingly, results from these two cohorts and our study all showed over-representation of *Fusobacterium* and *Parvimonas*. The Fn, Pm, Ps, Am four-bacterial panel combination seems to be unique for the Malaysian CRC population, compared to results from other studies.

In this study, we observed that metabolic functions involving DNA repair, ribosome activity and aminoacyl-tRNA biosynthesis were more abundant in the gut microbiome of CRC patients compared to non-CRC controls. It has been hypothesized that CRC-associated bacteria secretes toxins which will cause DNA damage in host cells and carcinogenesis^14^. Host cells subsequently respond by mounting an immune response via reactive oxygen species (ROS)-mediated pathways to eliminate the infecting bacteria^27^. In return, we suspect that the CRC gut microbiome would then require upregulation of pathways associated with DNA repair to survive the ROS attack. Protection of CRC-associated gut bacteria from the host immune system allows bacteria growth and further development of tumours in colon cells with the activation of ribosomal activity and aminoacyl-tRNA biosynthesis^28,29^. On another note, we observe slight elevation in polycyclic aromatic hydrocarbon (PAH) degradation metabolism in the CRC microbiome; incidentally, PAH metabolism is linked with red meat consumption and carcinogensis^30,31^.

Till date, various studies have been carried out to profile the CRC gut microbiome but there are differences in the approach including sequencing method and bioinformatics workflow ^32^. In our study, tumour / colonic mucosal tissue samples were used for DNA extraction, as these samples have been proposed, compared to stool samples, to give a more accurate picture of the gut microbiome landscape on tumour environments and enable functional studies into the role of these bacteria in tumorigenesis^33–36^. In addition, we were also able to perform species-level taxonomical classification for each microbiota using the One Codex bioinformatics platform. Many previous studies published only genera-level identification of gut microbiota using QIIME, a commonly-used bioinformatics pipeline. One Codex is a new pipeline using the assignment-first approach in taxonomical classification^37^, using the K-mer-based analysis which is also used by Kraken^38^ and CLARK^39^. At the time of our study, it is still not widely used for 16S rRNA gene analysis; nevertheless, we find the platform robust and user-friendly, allowing us to species-classify the gut microbiome of our study samples and further use these results for qPCR primer design in our validation phase of the study. In regards to this, the results from many earlier studies of CRC gut microbiome profiling were not further verified in a biological cohort^9,20,40–43^. In our study, bacteria found to be significantly abundant in the discovery phase CRC samples were also found to be over-represented in our CRC validation cohort. We showed the utility of qPCR for the validation of 16S rRNA gene sequencing results. We also found good AUROC, Sn, Sp, PPV and NPV values in our Pm-Ps-Fn-Am four-bacteria CRC biomarker panel, showing the importance of these bacteria in CRC.

There were some limitations in our study. The number of samples used in our study was smaller compared to other studies, but we showed that the results were reproducible in the validation cohort. On the other hand, due to limited numbers, bacteria identified as significantly different in various CRC demographics and specific to certain ethnicity, gender and CRC staging could not be further verified in our validation cohort. It is also unclear how these bacteria might contribute to the observed demographic-specific difference in abundance found in our study. In addition, at the time of study, due to unavailability of whole genome sequences of some bacteria found over-represented in our CRC patients, we could not design primers to validate their abundance using qPCR assay. Therefore, the over-representation of *Ruminococcus callidus*, *Eubacterium coprostanoligenes, Intestinimonas butyriciproducens* and *Ruminococcus bromii* in CRC remains to be confirmed. Some recent publications reported the possible role of these bacteria in gut health and pathology, but the number of reports were too few to allow definite conclusions^44–46^.

Furthermore, while usage of mucosal tissues in 16S rRNA gene sequencing enabled us to identify the gut microbiome present on tumours and deduce their roles in tumorigenesis, results from mucosal tissue sequencing might be discrepant from those found in stool samples of CRC patients. Therefore, the four-bacteria panel identified to be over-represented in tissues of our CRC patients could not be used for population screening of the disease. The currently available immunochemical fecal occult blood test (iFOBT) will be a more suitable method for this purpose. Nevertheless, iFOBT only has a 40% sensitivity to detect patients with advanced adenomas^47^. To this end, identification of over-abundant gut bacteria found in stool samples of advanced adenoma and early-stage CRC patients, and utilization of these bacteria as screening markers for early detection of CRC could be further explored.

## Conclusion

In summary, we identified Pm, Ps, Fn and Am as bacteria significantly abundant in our cohort of Malaysian CRC patients. The exact role of these bacteria in CRC initiation and progression remains to be investigated in further studies.

## Methodology

### Ethics statement, subject information and sample collection

This study was approved by the Universiti Kebangsaan Malaysia Research Ethics Committee (UKMREC) according to the declaration of the International Conference of Harmonization Good Clinical Practice Guideline (Ethics approval code: UKM 1.5.3.5/244/UMBI-2015-005). The study was carried out in two phases: discovery (gut microbiome profiling via 16S rRNA gene sequencing) and validation (qPCR amplification of 5 significant CRC-associated bacterial markers found in the discovery phase). Patients and controls for the discovery phase were recruited from those undergoing colonoscopy and tumour removal surgery at the Hospital Canselor Tuanku Muhriz, UKM Medical Centre (UKMMC), Kuala Lumpur, from 2015 to 2017. Written informed consent was obtained from each patient and control prior to colonoscopy. Subjects in this phase included individuals presenting with digestive symptoms and asymptomatic individuals undergoing colon screening. Exclusion criteria included history of any cancer or colon resection, subjects with gut diseases such as inflammatory bowel disease (IBD) and polyps, consumption of antibiotics for the past 3 months and unsuccessful colonoscopy procedure. Endoscopic pinch biopsies were performed from tumour sites, immediately flash frozen in liquid nitrogen after collection and stored at − 80˚C until further analysis. After colonoscopy results were obtained, subjects with confirmed CRC were grouped as “patients”, while subjects whose tissues contained no evidence of active gut pathology were grouped as “non-CRC”. For the validation phase, matched tissue samples were selected from the UMBI-UKMMC Biobank. Patients with recurrent CRC, history of other cancers, inflammatory bowel disease, and those who had either radiotherapy or chemotherapy prior surgery were excluded from this phase of the study. For controls, the corresponding samples from the discovery phase, together with archived control tissues from the UMBI-UKMMC Biobank were used.

### DNA extraction and 16S rRNA gene sequencing

Genomic DNA from flash frozen tissue samples was extracted using the QIAGEN DNA Micro Kit (discovery phase) and the Machery-Nagel NucleoSpin Tissue Kit (validation phase) as per manufacturers’ protocols. The quality of the extracted DNA was determined by gel electrophoresis and the Nanodrop 2000c. Amplicon libraries for the discovery phase were prepared according to Illumina’s 16S Metagenomic Sequencing Library Preparation protocol with some slight modifications. Briefly, amplicons were generated using primers targeting the V3/V4 region of 16S rRNA gene. PCR products were purified using E-Gel EX SizeSelect 2% agarose and subsequently attached with Nextera XT forward and reverse indices for barcoding. The amplicons were then purified using AMPure XP beads and quality-checked using the Agilent Bioanalyzer High Sensitivity DNA kit to determine library size. Libraries were quantified using Illumina Library Quantification kit (KAPA Biosystems) and normalized to 2 nM prior sequencing using a 2 × 250 bp MiSeq Reagent kit v2 on an Illumina MiSeq sequencer.

### Bioinformatics analyses

Sample de-multiplexing was performed using Illumina’s BCL2FASTQ algorithm by MiSeq Software Reporter. Raw FASTQ files were exported and processed by Trimmomatic v0.34^48^ for adapter trimming and quality filtering. Forward and reverse sequences of each sample were assembled using SeqPrep (https://github.com/jstjohn/SeqPrep) and converted to FASTA via FASTX-Toolkit (http://hannonlab.cshl.edu/fastx_toolkit/) prior to analysis using the QIIME v1.9.1^49,50^ and One Codex software^51^. In the QIIME analysis, assembled reads were clustered into Operational Taxonomic Units (OTUs) using UCLUST^52^ and aligned against the GreenGenes 16S rRNA gene database version 2013.05 (http://greengenes.lbl.gov) at 97% similarity threshold. Core diversity analyses were performed to determine alpha and beta-diversity of the samples. For species-level analysis, assembled FASTA files were uploaded to One Codex platform (https://www.onecodex.com/platform/) and aligned against the Targeted Loci database for species-level taxonomic classification.

LEfSe (Linear discriminant analysis effect size) was used to compare the relative abundance of different taxa between groups, where a *p*-value of less than 0.05 for the Kruskal–Wallis rank-sum test and a size-effect threshold of 2.0 on the logarithmic LDA score were applied for discriminative microbial biomarkers^53^. The gene functions of mucosal-associated microbiome for each group were predicted using Phylogenetic Investigation of the Communities by Reconstruction of Unobserved States (PICRUSt v1.1.3)^54^. After normalisation for 16S rRNA copy numbers, metagenomes were predicted based on KEGG (Kyoto Encyclopedia of Genes and Genomes) pathway database^55^ and summarised using KEGG from level 1, 2 and 3 metabolic functions. Differences in predicted metabolic function abundance between groups were identified using Statistical Analysis for Metagenomic Profile (STAMP)^56^. The STAMP software was used to assess significant statistical differences between the predicted metabolic function profiles using Welch’s t-test corrected for multiple-testing by Benjamini–Hochberg false discovery rate (FDR). Corrected *p*-values below 0.05 were considered significant.

### Quantitative PCR (qPCR)

qPCR was used to determine the relative abundance of candidate bacterial markers. Only bacterial species with available genome sequences for primer design were tested for relative abundance. Table 8 shows primers sequence of candidate markers designed using AlleleID v7.84 (PREMIER Biosoft, USA).

**Table 8:** List of primers used for qPCR validation.

Bacteria	Sequence (5′–3′)	Product size	Primer efficiency (%)
Total bacteria	F: GCAGGCCTAACACATGCAAGTC	324 bp	90.7
	R: CTGCTGCCTCCCGTAGGAGT		
*F. nucleatum*	F: CAACTTGGTGAGAACGAGGTATC	134 bp	103.7
	R: TGCTGGTGGTAGAGGTATGG		
*P. stomatis*	F: CGGCAGCAGGATACATAGC	136 bp	94.7
	R: TGGACAAGGAGTGGTAGGTT		
*A. muciniphila*	F: GAAGACGGAGGACGGAACT	126 bp	102.4
	R: GCGGATTGCTGACGAAGG		
*P. micra*	F: TCACAGTAGTCACAAGAGGAGAT	87 bp	103.5
	R: GGGAAGCATTGGCGGAAA		
*B. fragilis*	F: TTCATTGGGAAAAGTGTCGGTAT	65 bp	94.5
	R: GCATAGCATCATTCCGCTCTT		

All reactions were performed on a CFX96 Touch Real-time PCR Detection system in a 10µL reaction volume using SsoAdvanced Universal SYBR Green Super mix (Bio-Rad, USA). Each sample was assayed for 40 cycles in a triplicate reaction and relative abundance of each marker was calculated in reference to total bacteria DNA. Primers for total bacteria were adapted from a previous study^57^. Abundance of the tested bacterial markers was calculated as a relative unit normalised to the total bacteria of that sample, using the 2^−ΔΔCt^ method (where ΔΔCt = the average ΔCt value of each target—the average ΔCt value of total bacteria).

### Statistical analysis

Taxonomic differences of gut microbiome composition between CRC and control was analysed using the Mann–Whitney test, where a minimum fold change of > 1.5 in bacteria abundance and > 66% occurrence in CRC was considered as significantly enriched. To determine gut microbiome differences between different demographics, the Mann–Whitney test was also used, with a cut-off value of > 5 fold abundance. For CRC staging, Dukes’ B was classified as early CRC, while Dukes’ C and D were classified as advanced CRC. Spearman correlation coefficient analysis was used to investigate the correlation between bacterial candidate marker abundance detected from sequencing and qPCR techniques. Diagnostic value for the bacterial markers in identifying CRC patients were evaluated by calculating the area under the receiver-operating characteristic (ROC) curve. The best cut-off values were determined by ROC analyses from maximized Youden index and smallest distance value. Sensitivity and specificity values were compared to find the best panel combination that gives high positive predictive value (PPV) and negative predictive value (NPV). Logistic regression model was applied to obtain probability plot values for estimating the CRC incidence among all subjects. ROC curves were constructed from the logistic regression for four-bacteria panel data. All tests were performed by GraphPad Prism 7.0 or SPSS software v22.0. A nominal value of *p* < 0.05 was determined as statistical significance.

### Ethics approval and consent to participate

This study was approved by the National University of Malaysia Research Ethics Committee (UKMREC) according to the declaration of the International Conference of Harmonization Good Clinical Practice Guideline (Ethics approval code: UKM 1.5.3.5/244/UMBI-2015–005).

## Supplementary Information

Supplementary Dataset 1.

Supplementary Dataset 2.

## Data Availability

Data has been uploaded as Supplementary Dataset 1 and 2 of the manuscript.
